# Atopic Dermatitis and Psoriasis as Overlapping Syndromes

**DOI:** 10.1155/2020/7527859

**Published:** 2020-11-27

**Authors:** A. Bozek, M. Zajac, M. Krupka

**Affiliations:** ^1^Clinical Department of Internal Medicine, Dermatology and Allergology, Medical University of Silesia, Katowice, Poland; ^2^European Center for Diagnosis and Treatment of Urticaria (GA2LEN UCARE) in Zabrze, Medical University of Silesia, Katowice, Poland; ^3^Medical University of Wroclaw, Poland

## Abstract

**Methods:**

In this observational study, 38 children with concomitant AD and PS with a mean age of 6.5 ± 3.2 yrs were compared with 41 similar patients with AD only (5.3 ± 5.1 yrs) and 28 patients with PS only (6.4 ± 4.3 yrs). All patients underwent dermatological examinations, including determination of SCORAD and PASI scores. TNF-*α*, IFN-*γ*, IL-2, IL-4, IL-5, IL-6, IL-8, IL-12, IL-17, IL-18, IL-22, I:-33, and TARC/CCL17 were measured by ELISA according to the manufacturer's instructions.

**Results:**

Patients with concomitant AD and PS were frequently boys and overweight and had skin lesions equally distributed throughout the body. Children with concomitant AD and PS were more likely to report a family history of atopic disease than children with only AD or PS, and those with AD were more likely to report a family history of atopic disease than those with PS. Significant differences were observed in the concentration of IL-17 between patients with AD and PS and those with only AD or PS: 9.1 ± 3.7 pg/ml vs. 4.8 ± 2.9 pg/ml; and 9.1 ± 3.7 pg/ml vs. 5.2 ± 3.9 pg/ml, respectively (PD vs. AD, *p* = 0.01; PD vs. PS, *p* = 0.03).

**Conclusions:**

AD and PS can coexist. The role of T helper 17 cells may be more essential than believed.

## 1. Introduction

Atopic dermatitis (AD) and psoriasis (PS) are inflammatory skin disorders that are clinically different. However, in the young population, PS is commonly mistaken for AD [1]. AD affects 11%-30% of children, and PS affects 1%-3% of these children with AD **[**1, 2]. AD shows erythematous patches with excoriations associated with pruritus. PS presents with well-demarcated plaques covered with silvery-white scales [1, 3]. Despite the differences between AD and PS, they share some common features, such as infiltration of immune cells in the skin, altered expression of some proinflammatory cytokines, and barrier alterations [2, 4]. Although the pathogenesis of PS and AD is different, PS has been found to be associated with atopy and AD **[**3, 4]. However, PS can show eczematous changes in the acute phase, while AD can display psoriasiform lichenified changes in the persistent/resistant stage. Both AD and PS can be misinterpreted as the coexistence of two diseases [4, 5].

However, there is evidence that AD and PS can coexist as an overlapping syndrome: psoriasis-dermatitis [1]. This condition has various hypothetical origins [5, 6].

The aim of this study was to assess clinical characteristics and cytokine profiles in children with AD and concomitant PS and compare them with those in children with AD or PS.

## 2. Material and Methods

This was a prospective, observational, two-center study. This study included 38 children with a mean age of 6.5 ± 3.2 yrs with concomitant AD and PS. They were compared with 41 similar patients with AD only (5.3 ± 5.1 yrs) and 28 similar patients with PS only (6.4 ± 4.3 yrs).

Patients were eligible if they were diagnosed with AD and/or PS, between 2 and 10 yrs of age, had mild-to-severe AD symptoms according to the objective SCORing Atopic Dermatitis (SCORAD) index and/or mild-to-severe PS according to the objective PASI (Psoriasis Area Severity Index). Patients were observed for at least 12 months.

During the enrolment process, the diagnosis of AD was based on the patient's clinical features and historical characteristics according to the Hanifin and Rajka criteria [7]. Children with a single skin lesion were excluded. However, the final diagnosis of AD had to follow the UK working group criteria, which are specific for young children. In this way, AD was diagnosed when a child had itchy skin and minimum three of the following criteria: (1) visible flexural dermatitis involving the skin creases, such as the bends of the elbows or the areas behind the knees (or visible dermatitis on the cheeks and/or extensor areas in children aged 18 months or under); (2) a personal history of flexural dermatitis or dermatitis on the cheeks and/or extensor areas in children aged 18 months or under; (3) a personal history of dry skin in the last year; (4) a personal history of asthma or allergic rhinitis (or a history of atopic disease in a first-degree relative of children aged under 4 yrs of age); and (5) onset of signs and symptoms under the age of 2 yrs (this criterion was not used in children aged under 4 yrs) [1, 8].

PS was confirmed based on typical morphological characteristics (well-demarcated psoriatic plaques, guttate disease, acral fingertip eruptions, or napkin or pustular psoriasis) and history [1, 9, 10].

Patients with a diagnosis of concomitant AD and PS were observed for at least 12 months for confirmation. There were three independent dermatological assessments before cooccurrence of the diseases was diagnosed.

The exclusion criteria were as follows: receipt of oral corticosteroids or immunosuppressants prior to study entry; active skin disease other than AD and/or PS; other chronic diseases; and lack of written consent.

Overweight was defined as a body mass index (BMI) at or above the 85^th^ percentile according to the WHO.

## 3. Study Protocol

All patients underwent the following procedures: full dermatological examination including SCORAD and PASI score determination and procurement of blood samples for cytokine analysis. TNF-*α*, IFN-*γ*, IL-2, IL-4, IL-5, IL-6, IL-8, IL-12, IL-17, IL-18, IL-22, IL-33, and TARC/CCL17 were measured using ELISA according to the manufacturer's instructions (Thermo Fisher Scientific, US).

### 3.1. Statistical Analysis

Statistical analysis was performed using Statistica 8.2 (SaftPOl, Krakow, Poland). ANOVA, the Wilcoxon test, and Student's *t*-test were performed to compare relevant variables. A *p* value < 0.05 was considered significant.

## 4. Results

The characteristics of the study groups are presented in Table 1. Patients with concomitant AD and PS were frequently boys and overweight and frequently had skin lesions equally distributed throughout the body. Children with concomitant AD and PS had a family history of atopic disease more often than those with AD or PS only, and those with AD only had a family history of atopic disease more often than those with PS only.

### 4.1. Cytokine Profiling

Patients with concomitant AD and PS had significantly higher serum concentrations of IL-17 than patients with AD or PS only, and patients with AD only had significantly higher serum concentrations of IL-22 than the other groups. The cytokine profiles are shown in Table 2.

## 5. Discussion

Cooccurrence of AD and PS is not common, and there is little information about the clinical course of patients with both these diseases. The coexistence of these diseases in the same person may be due to at least three scenarios: (1) disease cooccurrence and flare-up at the same time, which is truly rare; (2) disease cooccurrence but alternating flare-ups (that is, when one flares-up, the other subsides, or vice versa), which can be occasionally observed; and (3) cooccurrence at different life stages (for example, atopic dermatitis may present in childhood, then disappear for years, followed by the development of psoriasis later in adulthood). The third is probably the most common scenario [5]. In the present cohort of patients with concomitant AD and PS, the patients mainly matched the first and sometimes the second scenario. Other studies have found similar results [5, 6].

The obtained results revealed clinical and immunological differences between the analysed groups.

The pathophysiology of AD and PS is complex. First, defects in epidermal barrier function are crucial to disease initiation; however, many secondary mechanisms are observed. Dysfunction of the skin barrier increases the penetration of allergens and microorganisms into the skin. These antigens induce immunological responses, for example, via T helper 2 and T helper 22 cells and proinflammatory cytokines, in the acute phase. This leads to skin inflammation. Then, during the progression of AD, T helper 1 cells may play a role [11, 12]. Keratinocyte dysfunction is of major importance in the pathomechanism of psoriasis. T helper 1 cell overactivation induces psoriasis; however, T helper 17 cells have been demonstrated to be critical for this disease. T helper 17 cells produce cytokines, which affect keratinocyte proliferation and stimulate inflammation [13, 14]. The obtained results indicate IL-17, and therefore, the participation of T helper 17 cells may be especially crucial in concomitant AD and PS. However, in the study, the group with psoriasis had IL-17 values significantly lower than those in the other groups, despite the evidence of the important role of Th17 cells in this disease [11, 12]. This discrepancy could have been the results of the small sample size or mild forms of disease in the PS group. However, the PS group did have lower levels of IL-4, IL-5, and IL-22 than the other groups, which suggests that patients in the PS group have different mechanisms of diseases compared with the patients in the other groups. The cytokine profile of the AD group agreed with the known AD mechanism [15]. Some relatively new biomarkers of AD, such as IL-33 and TARC, were elevated in the group with concomitant AD with PS and the AD group compared to the PS group, but the differences were not significant. These results may indicate a large variety in the scope of AD and PS subtypes, which is currently being studied [16].

Patients with concomitant AD and PS had a similar distribution of the affected skin areas. The results of this study are largely consistent with the observations of other authors [5]. However, an important finding is the patients in the concomitant AD and PS group were mostly boys and overweight. This finding is not in line with the data reported by Docampo showing that overweight is characteristic of patients with PS [5]. Results related to overweight can be influenced by many factors: group selection, size of the study group, environmental conditions, and eating habits. Whether obesity is a risk factor for concomitant AD and PS remains an open question that requires testing in a larger group of patients. The male dominance seen in this study was consistent with previous observations [5, 6].

There are some limitations of the study: relatively small study group (but similar to the sample size of other studies), lack of biopsy data, and a relatively short follow-up duration (only 12 months). Unfortunately, it is not ethically feasible to perform biopsies in very young children. However, in the few children who underwent biopsies of skin lesions, the histopathological findings in the biopsy samples were not different from those discovered in the clinical assessment of these patients. These data were not included because they came from a small number of patients. Moreover, distinguishing between AD and PS based on single skin changes alone can be difficult. Psoriasiform reactions are sometimes observed in patients with AD, and eczematous reactions are possible in patients with psoriasis, for example, when biologic drugs are administered [17, 18].

## 6. Conclusion

We agree with the hypothesis of other authors that AD and psoriasis are separate entities of a disease spectrum, and overlapping disease characteristics can be seen. The role of T helper 17 cells may be more meaningful than originally believed.

## Figures and Tables

**Table 1:** Characteristics of the study groups.

	AD&PS *n* = 38	AD *n* = 41	PS *n* = 28
Mean age (yrs)	6.5 ± 3.2	5.3 ± 5.1	6.4 ± 4.3
Female (%)	13 (34)^^^	23 (56)	18 (64)
Other atopic disease			
2003BA (%)	3 (8)	8 (20)^∗^	0
AR (%)	6 (16)	17 (41)^∗^	6 (21)
Family history			
ADEA (%)	6 (15)	18 (43)^^	4 (14)
PS (%)	11 (29)	2 (5)^#^	7 (25)
Affected area			
Skin			
Head	11 (29)	15 (24)	11 (39)
Arms	10 (26)	15 (36)	8 (29)
Legs	9 (24)	13 (31)	9 (32)
Trunk	14 (37)	8 (19)^∗∗^	11 (39)
Nails	9 (24)	14 (34)	9 (32)
SCORAD	35 ± 8	42 ± 11	—
PASI	28 ± 10	—	34 ± 9
BMI >23	14 (37)^^^^	5 (12)	7 (25)
Confirmed arthritis	4 (11)	4 (10)	5 (19)

Legend: AD&PS: psoriatic dermatitis; ADEA: atopic disease; PS: psoriasis; BA: allergic bronchial asthma; AR: allergic rhinitis; SCORAD: SCORing Atopic Dermatitis; PASI: psoriasis area and severity index; ^: significant male dominance in AD&PS vs. AD and AD&PS vs. PS (Wilcoxon test, *p* < 0.05); ^∗^ increased frequency of BA and AR in AD vs. AD&PS and AD vs. PS (*p* < 0.05); ^^^^ increased family history of atopic disease in AD vs. AD&PS and AD vs. PS (*p* < 0.05); ^#^ decreased family history of PS in AD vs. the other groups (*p* < 0.05); ^∗∗^ decreases skin of trunk involvement in AD vs. the other groups (*p* = 0, 05); ^^^^ overweight was more common in the AD&PS group than in the other groups (*p* < 0.05).

**Table 2:** Levels of tested cytokines in blood serum (values are the mean ± SD).

	AD&PS *n* = 38	AD *n* = 41	PS *n* = 28
TNF-*α* (pg/mL)	18.9 ± 10.2	13.9 ± 9.2	14 ± 6.9
IFN-*γ* (IU/mL)	0.45 ± 0.13	0.3 ± 0.11	0.5 ± 0.29
IL-2 (pg/mL)	39.2 ± 13.6	44.5 ± 14.9	41.8 ± 9.5
IL-4 (pg/mL)	51.7 ± 33.2	56 ± 28.1	34.9 ± 10.3^
IL-5 (pg/mL)	187 ± 96	219 ± 102	75 ± 45^
IL-6 (pg/mL)	11.9 ± 7.2	9.4 ± 4.2	8.9 ± 3.3
IL-8 (pg/mL)	28.8 ± 10.3	22.8 ± 11.6	25.2 ± 8.4
IL-12 (pg/mL)	32.1 ± 12.7	29 ± 10.3	27.8 ± 12
IL-17 (pg/mL)	10.1 ± 3.7^∗^	4.8 ± 2.9	5.2 ± 3.9
IL-18 (pg/mL)	67.4 ± 21.3	70.1 ± 22.9	66.4 ± 18.3
IL-22 (pg/mL)	678 ± 262	1394 ± 341^∗∗^	542.4 ± 210
IL-33 (pg/mL)	89.7 ± 24.7	102 ± 64.1	7 ± 43.7
TARC/CCL17	145 ± 62	195 ± 87	132 ± 42

Legend: AD&PS: psoriatic dermatitis; ADEA: atopic disease; PS: psoriasis; ^^^ increased mean serum concentration in PS vs. AD&PS and PS vs. AD (Student's *t*-test, *p* < 0.05); ^∗^ increased mean serum concentration in AD&PS vs. AD and AD&PS vs. PS (*p* < 0.05); ^∗∗^ increased mean serum concentration in AD vs. AD&PS and AD vs. PS (*p* < 0.05).

## Data Availability

The data used to support the findings of this study were supplied by prof. Jerzy Jarzab under license and so cannot be made freely available. Requests for access to these data should be made to prof. Jerzy Jarzab email: dermalerg@sumedu.pl
